# Impact of cardiovascular risk factors on myocardial work—insights from the STAAB cohort study

**DOI:** 10.1038/s41371-021-00509-4

**Published:** 2021-03-02

**Authors:** Floran Sahiti, Caroline Morbach, Vladimir Cejka, Theresa Tiffe, Martin Wagner, Felizitas A. Eichner, Götz Gelbrich, Peter U. Heuschmann, Stefan Störk

**Affiliations:** 1 Comprehensive Heart Failure Center, University Hospital and University of Würzburg, Würzburg, Germany; 2 Department of Medicine I, University Hospital Würzburg, Würzburg, Germany; 3 Institute of Clinical Epidemiology and Biometry, University of Würzburg, Würzburg, Germany; 4 Clinical Trial Center, University Hospital Würzburg, Würzburg, Germany

**Keywords:** Echocardiography, Risk factors

## Abstract

Myocardial work is a new echocardiography-based diagnostic tool, which allows to quantify left ventricular performance based on pressure–strain loops, and has been validated against invasively derived pressure–volume measurements. Myocardial work is described by its components (global constructive work [GCW], global wasted work [GWW]) and indices (global work index [GWI], global work efficiency [GWE]). Applying this innovative concept, we characterized the prevalence and severity of subclinical left ventricular compromise in the general population and estimated its association with cardiovascular (CV) risk factors. Within the Characteristics and Course of Heart Failure STAges A/B and Determinants of Progression (STAAB) cohort study we comprehensively phenotyped a representative sample of the population of Würzburg, Germany, aged 30–79 years. Indices of myocardial work were determined in 1929 individuals (49.3% female, mean age 54 ± 12 years). In multivariable analysis, hypertension was associated with a mild increase in GCW, but a profound increase in GWW, resulting in higher GWI and lower GWE. All other CV risk factors were associated with lower GCW and GWI, but not with GWW. The association of hypertension and obesity with GWI was stronger in women. We conclude that traditional CV risk factors impact selectively and gender-specifically on left ventricular myocardial performance, independent of systolic blood pressure. Quantifying active systolic and diastolic compromise by derivation of myocardial work advances our understanding of pathophysiological processes in health and cardiac disease.

## Introduction

**Fig. 1:**
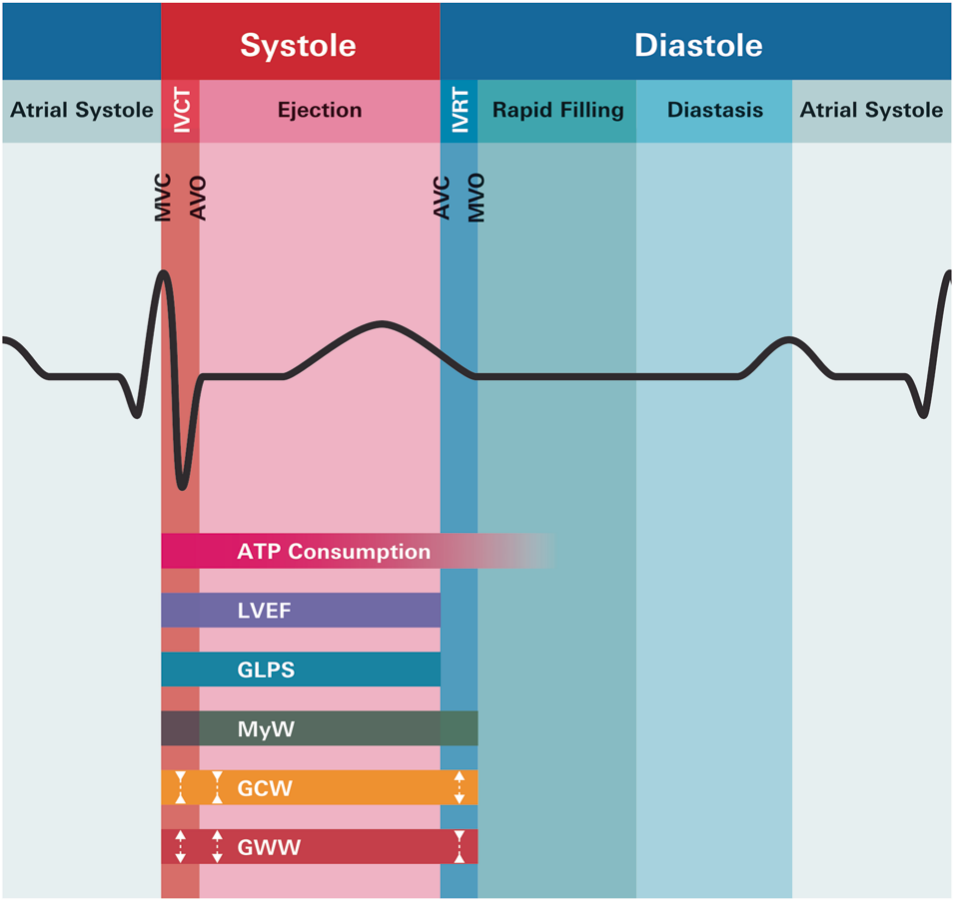
Surrogate measures of left ventricular function in relation to the cardiac cycle and ATP consumption. Myocardial work includes total active myocardial work and allows us to differentiate constructive from wasted work components. LVEF left ventricular ejection fraction, GLPS global longitudinal peak strain, MyW myocardial work, GCW global constructive work, GWW global wasted work, MVC mitral valve closure, AVO aortic valve opening, AVC aortic valve closure, MVO mitral valve opening, IVRT isovolumic relaxation time, IVCT isovolumic contraction time, ATP adenosine triphosphate.

Myocardial work (MyW) is a novel echocardiographic method allowing to noninvasively determine total active myocardial performance via its two components constructive and wasted MyW (Fig. 1) [1]. Following known concepts of estimation of left ventricular (LV) systolic function by afterload-adjusted parameters of fiber shortening [2], the derivation of MyW integrates information on myocardial deformation (by speckle-tracking longitudinal strain) and afterload by pressure–strain loops (PSL). As such, the energy-consuming phases of the cardiac cycle, i.e., systolic and early diastolic active MyW can be quantified (Fig. 1). This information can be derived segment-by-segment or expressed as a global value, i.e., global constructive and wasted MyW (GCW, GWW). Echocardiography-derived parameters of MyW showed a high correlation with invasive validation measurements [1, 3, 4]. MyW demands the imputation of systemic blood pressure; it is markedly less load-dependent compared to conventional measures of LV function as ejection fraction and global longitudinal strain (GLPS) [1, 5, 6], and might thus overcome the disadvantage of these measures of overestimating LV dysfunction in individuals with increased afterload [6, 7]. MyW might therefore be superior in detecting “real” subclinical LV dysfunction. Further, the derived PSL area was shown to reliably reflect the myocardial metabolic demand and oxygen consumption [1], thus, also providing insights into myocardial energetics.

Cardiovascular (CV) risk factors increase the risk to develop CV disease both indirectly, i.e., by altering the metabolic environment and homeostasis, or directly, i.e., by adversely affecting myocardial function [8]. As such, CV risk factors also associate with subclinical alterations in systolic and diastolic function and might accelerate conditions preceding heart failure with reduced and/or preserved ejection fraction [9–11]. Consistent with this view, changes of LV structure and function over time showed a more favorable pattern in the absence of CV risk factors [12].

Because the invasive assessment of pressure–volume loops is restricted to smaller patient samples due to the limited availability and the potential risks of an invasive procedure, echocardiography-based assessment of MyW is a readily available and noninvasive method. In the present analysis, we aimed to investigate the association between CV risk factors with MyW and its components in a large, well-characterized cohort derived from the general population.

## Methods

### Population

The Characteristics and Course of Heart Failure STAges A/B and Determinants of Progression (STAAB) cohort study recruited and characterized a representative sample of the population of Würzburg, Germany, aged 30–79 years, free of symptomatic heart failure at inclusion. Details of the study design have been published previously [13]. The STAAB study complies with the Declaration of Helsinki and was approved by the Ethics Committee of the Medical Faculty, University of Würzburg (J-117.605-09/13). All participants provided written informed consent prior to any study examination. For the present analysis, we concentrated on the first half of the STAAB study population (*n* = 2473), which, due to a planned interim analysis [13], met sex and age stratification criteria of the total sample.

### Baseline examination

Blood pressure (sitting position after 5 min of rest), body height and weight, smoking habits, and current medication were assessed according to standard operating procedures [13]. Laboratory measurements were performed at the Central Laboratory, University Hospital Würzburg, including fasting lipid profile, estimated glomerular filtration rate (eGFR), glycosylated haemoglobin (HbA1c), and plasma glucose levels.

CV risk factors included in the following analysis were *obesity* (body mass index > 30 kg/m^2^) [14], *dyslipidaemia* (low-density lipoprotein [LDL] > 190 mg/dl [15], or on lipid-lowering medication), *diabetes mellitus* (HbA1c > 6.5%, or fasting plasma glucose > 7 mmol/l, or plasma glucose 2 h after oral intake of 75 g glucose > 11.1 mmol/l [16] or on antidiabetic medication), *hypertension* (blood pressure ≥ 140/90 mmHg [17] or on antihypertensive therapy), and *smoking* (current smoker or ex-smoker).

### Echocardiography and quality assurance

All patients underwent standard transthoracic echocardiography using Vivid S6^®^ (M4S Sector Array Transducer operating at 1.5–4.3 MHz, GE Healthcare, Horten, Norway). Two-dimensional images from the LV apical four-, two-, and three-chamber views were recorded with a frame rate of 50–80 s^−1^ and stored digitally. Image acquisition was performed by trained and certified sonographers on one echocardiography machine with consistent system presets according to a prespecified protocol. The characteristics and effectiveness of performance measures of the echocardiography quality assurance program have been published previously [18]. MyW analysis was performed by one researcher (F.S.). For assessment of observer variability, 20 randomly selected scans were read by the same observer twice, 2 weeks apart, for interobserver variability, the same scans were read by a second person (C.M.) blinded to the previous results. The inter-and intraobserver variability regarding MyW parameters was favorably low (Supplemental Table S1).

### Analysis of myocardial work

For MyW analysis, a previously generated empiric normalized reference curve for LV pressure was used [1]. This reference curve was adjusted (a) by aligning valvular times as assessed by echocardiography and (b) by including blood pressure measured by cuff manometer as a surrogate of peak systolic LV pressure. Aortic and mitral valve closure and opening times were assessed by CW Doppler through the aortic valve and PW Doppler of the mitral valve. However, as the changes in heart rate during the examination would possibly affect the loop area, these time points were visually verified in the apical three-chamber view and manually adjusted where necessary. Two-dimensional images from the LV apical four-, two-, and three-chamber views were analyzed off-line using Automated Functional Imaging (EchoPAC^®^, Version 202, GE) to determine GLPS. Once GLPS was determined, the final adjustments for valve opening and/or closure times were done. We further provided the program with blood pressure values, to facilitate the derivation of the following parameters: GCW-mmHg%, i.e., work performed during shortening in systole and adding negative work during lengthening in isovolumic relaxation, also defined as work contributing to pump function; global wasted work (GWW-mmHg%), i.e., work performed during lengthening in systole or work performed during shortening against a closed aortic valve in isovolumic relaxation; global work index (GWI-mmHg%), i.e., the total amount of work within the pressure-strain loop area calculated from mitral valve closure to mitral valve opening; GWE-%, i.e., GCW/(GCW + GWW). All indices were calculated as the mean of respective segmental values (18-segment model). Myocardial work was measured as detailed in a previous report of our research group [19]. We excluded subjects from further analysis if more than one LV segment was unsuitable for analysis due to poor tracking or suboptimal image quality. The latter was defined according to the American Society of Echocardiography as the inability to detect two or more contiguous segments in any of the three apical windows [20].

### Data analysis

Statistical analysis was performed using SPSS (Version 25, SPSS Inc., Chicago, USA). Descriptives of continuous variables are provided as means (standard deviation), and categorical variables are presented as frequencies (percent). The variables were assessed for normality using the Shapiro–Wilk test. Differences in normally distributed variables were assessed using the *t* test. Non normal distributed variables were assessed using the Mann–Whitney *U* test. The relationship of MyW domains with sex, age, and CV risk factors was examined by analysis of covariance (ANCOVA). Chi-square tests were used to compare categorical variables. Observer variability was assessed using Bland–Altman 95% limits of agreement. First, a univariable model for each CV risk factor was built and in a second step adjusted for age and sex. Furthermore, to examine the association of the CV risk factors with MyW indices independent of blood pressure, we additionally adjusted for systolic blood pressure. Then, interaction for sex was tested, and, in case of statistical significance, respective values for men and women were reported. All tests were performed two-sided. *P* values < 0.05 were considered statistically significant.

## Results

The sample of the first-planned interim analysis comprised 2473 individuals. A total of *n* = 1929 individuals (mean age 54 ± 12 years, 49.3% women) entered the present analysis. Reasons to exclude *n* = 544 participants were the following: significant non-myocardial heart disease, *n* = 47 (not in sinus rhythm, more than mild regurgitation, any stenosis of the mitral or aortic valve, symptomatic heart failure); missing blood pressure value, *n* = 16; technical issues regarding the required views, *n* = 143; suboptimal image quality, *n* = 338. Comparing analyzed vs. excluded participants in a sensitivity analysis showed that non-analyzable subjects were more often female, slightly older, and exhibited a slightly worse clinical CV risk profile across the entire spectrum (Supplemental Table S2).

A subgroup of 439 participants (23%) exhibited no CV risk factor. By contrast, 1490 participants (77%) had at least one CV risk factor. Compared to the subgroup of participants with no CV risk factors, participants with ≥1 CV risk factor were older, more often male, and had higher body mass index and less negative GLPS. There was no difference in LV ejection fraction (LVEF) between groups. Compared to women, men had lower LVEF and, consistently, less negative GLPS. Further, men exhibited higher body mass index, plasma glucose level, systolic blood pressure, and more often hypertension, diabetes, and dyslipidaemia. There was no sex-specific difference in the prevalence of smoking or obesity (Table 1).

**Table 1:** Baseline characteristics of the study population.

	Total sample	No CV risk factor	≥1 CV risk factor	*P* value	Women	Men	*P* value
	*n* = 1929	*n* = 439	*n* = 1490		*n* = 951	*n* = 978	
Female sex	951 (49.3)	248 (56.4)	703 (47.1)	0.001	–	–	–
Age [years]	54 (12)	49 (11)	55 (11)	<0.001	53 (11)	54 (12)	0.151
Body mass index [kg/m^2^]	26 (4)	24 (3)	27 (4)	<0.001	25 (5)	27 (4)	<0.001
LVEF Simpson [%]	61 (4)	61 (4)	60 (5)	0.111	61 (4)	60 (4)	<0.001
GLPS [-%]	21 (3)	21 (2)	20 (4)	<0.001	22 (4)	20 (2)	<0.001
LVEDV	99 (25)	97 (25)	100 (25)	0.062	86 (19)	113 (23)	<0.001
LVESV	39 (11)	38 (10)	40 (12)	<0.001	33 (9)	45 (11)	<0.001
LV mass [g]	138 (39)	121 (32)	144 (39)	<0.001	116 (28)	160 (36)	<0.001
MV E wave [m/s]	0.7 (0.1)	0.7 (0.1)	0.7 (0.2)	0.011	0.74 (0.1)	0.67 (0.1)	<0.001
MV A wave [m/s]	0.6 (0.2)	0.5 (0.1)	0.6 (0.2)	<0.001	0.63 (0.2)	0.60 (0.2)	0.004
Mean E’ [m/s]	0.09 (0.03)	0.11 (0.02)	0.09 (0.03)	<0.001	0.09 (0.02)	0.10 (0.3)	<0.001
E/E’	8 (3)	7 (2)	8 (3)	<0.001	8 (3)	7 (2)	<0.001
IVRT [ms]	94 (18)	89 (14)	95 (18)	<0.001	90 (15)	96 (19)	<0.001
Total cholesterol [mg/dl]	208 (38)	202 (34)	209 (39)	0.001	211 (39)	204 (37)	<0.001
LDL cholesterol [mg/dl]	122 (34)	117 (30)	124 (35)	<0.001	121 (35)	124 (33)	0.059
eGFR [mL/min]	87 (15)	90 (14)	86 (15)	<0.001	87 (15)	87 (14)	0.475
HbA1c [%]	5.5 (0.6)	5.3 (0.3)	5.5 (0.6)	<0.001	5.5 (0.6)	5.5 (0.6)	0.435
Glucose value [mmol/L]	5.5 (0.9)	5.2 (0.3)	5.6 (1.0)	<0.001	5.4 (0.8)	5.7 (1.0)	<0.001
Glucose value after 2 h [mmol/L]	6.0 (1.7)	5.6 (1.3)	6.1 (1.8)	<0.001	6 (1.6)	6 (1.8)	0.511
Systolic blood pressure [mmHg]	130 (18)	122 (11)	133 (18)	<0.001	127 (19)	134 (16)	<0.001
Diastolic blood pressure [mmHg]	78 (10)	75 (7)	80 (10)	<0.001	77 (10)	80 (9)	<0.001
Hypertension	849 (44)	–	849 (44)	–	369 (39)	480 (49)	<0.001
Diabetes	157 (8)	–	157 (8)	–	59 (6)	98 (10)	0.002
Smoking	376 (19)	–	376 (19)	–	170 (18)	206 (21)	0.075
Obesity	302 (16)	–	302 (16)	–	140 (15)	162 (17)	0.266
Dyslipidaemia	255 (13)	–	255 (13)	–	106 (11)	149 (15)	0.008

Data are *n* (%) or mean (SD) or median (interquartiles).

*CV* cardiovascular, *LVEF* left ventricular ejection fraction, *GLPS* global longitudinal peak strain, *LVEDV* left ventricular end-diastolic volume, *LVESV* left ventricular end-systolic volume, *MV* mitral valve, *IVRT* isovolumic relaxation time, *LDL* low-density lipoprotein, *eGFR* estimated glomerular filtration rate, *HbA1c* glycosylated haemoglobin.

### Myocardial work indices

In the group with ≥1 CV risk factor we found markedly higher GWW, but only slightly higher corresponding GCW. Thus, the complementary ratios GWI and GWE were changed accordingly, i.e., increased GWI and decreased GWE, when compared to the subgroup with no CV risk factors. We observed significant yet minor differences between men and women, 3% for GCW and 5% for GWI, respectively. Further, in both subgroups with and without CV risk factors, women had significantly higher GWI when compared to men, whereas no such sex-related difference was apparent for GWW (Table 2).

**Table 2:** Myocardial work characteristics of the study population according to risk factors and sex.

	Total sample	No CV risk factor	≥1 CV risk factor	*P* value	Women	Men	*P* value
	*n* = 1929	*n* = 439	*n* = 1490		*n* = 951	*n* = 978	
GCW [mmHg%]	2505 (428)	2440 (334)	2526 (459)	<0.001	2550 (439)	2465 (427)	<0.001
GWW [mmHg%]	82 (58–119)	75 (54–109)	85 (60–123)	<0.001	83 (60–120)	82 (57–118)	0.685
GWI [mmHg%]	2277 (396)	2224 (310)	2293 (416)	0.001	2340 (408)	2216 (373)	<0.001
GWE [%]	96 (95–97)	96 (95–97)	96 (94–97)	<0.001	96 (95–97)	96 (94–97)	0.093

Data are *n* (%) or mean (SD) or median (interquartiles).

*CV* cardiovascular, *GCW* global constructive work, *GWW* global wasted work, *GWI* global work index, *GWE* global work efficiency.

### Association of CV risk factors with myocardial work indices

#### Global constructive work

GCW was positively associated with systolic blood pressure, cholesterol, glucose level after 2 h, hypertension, and inversely associated with body mass index, obesity, and smoking (Table 3). After adjustment for age and sex, GCW was associated with systolic blood pressure, glucose level, and hypertension. Higher body mass index, diabetes, smoking, obesity, and dyslipidaemia were significantly associated with lower GCW. After additional adjustment for systolic blood pressure, GCW remained inversely associated with body mass index, obesity, HbA1c, glucose level after 2 h, diabetes mellitus, smoking, and dyslipidaemia. Further, the association of GCW with obesity was more pronounced in women when compared to men.

**Table 3:** Quantitative impact of CV risk factors on global constructive work (GCW), measured in mmHg%.

	Univariable model		Interaction with sex	Adjusted for age and sex			Adjusted for age, sex, systolic blood pressure	
	Δ (95% CI)	*P* value	*P* value	Sex	Δ (95% CI)	*P* value	Δ (95% CI)	*P* value
Body mass index [kg/m^2^]	−7 (−11 to −2)	0.004	0.005	M	−17 (−24 to −10)^a^	<0.001	−19 (−22 to −16)	<0.001
				F	−4 (−10 to +2)	0.154		
Systolic blood pressure [mmHg]	+17 (+16 to +17)	<0.001	0.376	All	+18 (+18 to +19)	<0.001	–	–
Total cholesterol [mg/dl]	+0.9 (+0.3 to +1)	0.001	0.466	All	+0.3 (−0.2 to +0.8)	0.296	–	–
LDL cholesterol [mg/dl]	+0.3 (−0.3 to +0.8)	0.319	0.420	All	−0.09 (−0.6 to +0.5)	0.758	–	–
HbA1c [%]	+11 (−24 to +45)	0.552	0.272	All	−59 (−95 to −23)	0.001	−97 (−122 to −72)	<0.001
Glucose level [mmol/L]	+4 (−15 to +24)	0.662	0.493	All	−11 (−31 to +9)	0.226	–	–
Glucose level after 2 h [mmol/L]	+36 (+23 to +49)	<0.001	0.685	All	+25 (+12 to +39)	<0.001	M: +3 (−9 to +16)	0.603
				All			F: −17 (−31 to −2)^b^	0.028
Diabetes	−13 (−83 to +57)	0.718	0.312	All	−80 (−150 to −11)	0.024	−161 (−210 to −113)	<0.001
Hypertension	+303 (+267 to +339)	<0.001	0.143	All	+290 (+250 to +331)	<0.001	–	–
Smoking	−186 (−234 to −139)	<0.001	0.968	All	−160 (−206 to −113)	<0.001	–69 (−102 to −36)	<0.001
Obesity	−99 (−151 to −46)	<0.001	0.527	All	−123 (−174 to −72)	<0.001	M: −137 (−185 to −89)^b^	<0.001
							F: −229 (−281 to −178)	<0.001
Dyslipidaemia	−16 (−73 to +40)	0.573	0.758	All	−98 (−155 to −41)	0.001	−94 (−134 to −54)	<0.001

Estimates were derived from ANCOVA models and report the strength of association of individual CV risk factors (per unit) on GCW as absolute difference with respective 95% confidence interval. E.g., a positive increment in body mass index of 1 kg/m^2^ was associated with a 7 mmHg% decrement of GCW. Sex-specific associations are reported only ifinteraction with sex yielded a *p* value < 0.05.

^a^Interaction with sex was statistically significant after adjusting for age and sex.

^b^After adjustment for systolic blood pressure, there was a significant interaction with sex. *LDL* low-density lipoprotein, *HbA1c* glycosylated haemoglobin.

#### Global wasted work

GWW was positively associated with systolic blood pressure, hypertension, cholesterol, dyslipidaemia, diabetes mellitus, and glucose level. After adjustment for age and sex, systolic blood pressure and hypertension remained associated with higher GWW (Table 4).

**Table 4:** Quantitative impact of CV risk factors on global wasted work (GWW), measured in mmHg%.

	Univariable model		Interaction with sex	Adjusted for age and sex		
	Δ (95% CI)	*P* value	*P* value	Sex	Δ (95% CI)	*P* value
Body mass index [kg/m^2^]	+0.3 (−0.3 to +0.8)	0.329	0.558	All	−0.4 (−0.9 to +0.2)	0.165
Systolic blood pressure [mmHg]	+1.0 (+0.9 to +1.1)	<0.001	0.643	All	+0.9 (+0.8 to +1.0)	<0.001
Total cholesterol [mg/dl]	+0.09 (+0.03 to +0.1)	0.004	0.598	All	+0.01 (−0.05 to +0.07)	0.646
LDL cholesterol [mg/dl]	0.06 (−0.01 to +0.1)	0.099	0.608	All	−0.007 (−0.07 to +0.06)	0.826
HbA1c [%]	+11 (+6 to +15)	<0.001	0.785	All	+1 (−3 to +6)	0.537
Glucose level [mmol/L]	+5 (+3 to +7)	<0.001	0.283	All	+2 (−0.4 to +4)	0.107
Glucose level after 2 h [mmol/L]	+2 (+1 to +4)	0.003	0.379	All	0.8 (−0.7 to +2)	0.293
Diabetes	+13 (+5 to +21)	0.002	0.594	All	+2 (−7 to +10)	0.705
Hypertension	+29 (+25 to +33)	<0.001	0.671	All	+20 (+16 to +25)	<0.001
Smoking	−4 (−10 to +2)	0.175	0.115	All	−0.2 (−6 to +5)	0.935
Obesity	+6 (−0.2 to +12)	0.059	0.938	All	+2 (−4 to +8)	0.518
Dyslipidaemia	+12 (+5 to +18)	0.001	0.260	All	−1 (−8 to +6)	0.752

For interpretation of estimates and abbreviations please refer to the legend of Table 3.

#### Global work index

GWI was inversely associated with body mass index, obesity, HbA1c, smoking, and positively associated with systolic blood pressure, hypertension, cholesterol, glucose level after 2 h (Table 5). After adjustment for age and sex, GWI was inversely associated with body mass index (men only), obesity, HbA1c, smoking, dyslipidaemia, and positively associated with glucose level after 2 h, systolic blood pressure, hypertension (the association was more pronounced in women). After additional adjustment for systolic blood pressure, an inverse association with body mass index (either sex), HbA1c, obesity, dyslipidaemia, and smoking remained.

**Table 5:** Quantitative impact of CV risk factors on global work index (GWI), measured in mmHg%.

	Univariable model		Interaction with sex	Adjusted for age and sex			Adjusted for age, sex, systolic blood pressure	
	Δ (95% CI)	*P* value	*P* value	Sex	Δ (95% CI)	*P* value	Δ (95% CI)	*P* value
Body mass index [kg/m^2^]	−5 (−9 to −1)	0.019	0.002	M	−13 (−19 to −7)^a^	<0.001	–14 (–17 to –11)	<0.001
				F	−0.1 (−5 to +5)	0.968		
Systolic blood pressure [mmHg]	+14 (+14 to +15)	<0.001	0.877	All	+17 (+16 to +17)	<0.001	–	–
Total cholesterol [mg/dl]	+0.8 (+0.3 to +1)	0.001	0.862	All	+0.3 (−0.1 to +0.8)	0.163	–	–
LDL cholesterol [mg/dl]	+0.2 (−0.3 to +0.7)	0.481	0.794	All	−0.005 (−0.5 to +0.5)	0.984	–	–
HbA1c [%]	−2 (−34 to +30)	0.917	0.435	All	−46 (−79 to −12)	0.008	–80 (–104 to –56)	<0.001
Glucose level [mmol/L]	−3 (−21 to +16)	0.776	0.974	All	−8 (−27 to +10)	0.383	–	–
Glucose level after 2 h [mmol/L]	+31 (+18 to +43)	<0.001	0.985	All	+24 (+12 to +37)	<0.001	–	–
Diabetes	−20 (−84 to +45)	0.552	0.696	All	−53 (−118 to +12)	0.108	–	–
Hypertension	+253 (+219 to + 287)	<0.001	0.046	M	+240 (+190 to +288)^a^	<0.001	–	–
				F	+307 (+256 to +358)	<0.001		
Smoking	−160 (−204 to −116)	<0.001	0.827	All	−140 (−184 to −97)	<0.001	−58 (−90 to −26)	<0.001
Obesity	−82 (−131 to −34)	0.001	0.349	All	−95 (−143 to −48)	<0.001	M: −115 (−161 to −69)^b^	<0.001
							F: −184 (−233 to −134)	<0.001
Dyslipidaemia	−30 (−82 to +22)	0.256	0.908	All	−77 (−130 to −23)	0.005	−73 (−111 to −35)	<0.001

For interpretation of estimates and abbreviations please refer to the legend of Table 3.

^a^Interaction with sex was statistically significant after adjusting for age and sex.

^b^After adjustment for systolic blood pressure, there was a significant interaction with sex.

#### Global work efficiency

GWE was inversely associated with systolic blood pressure, hypertension, diabetes, HbA1c, glucose level, body mass index, obesity, cholesterol, and dyslipidaemia (Table 6). After adjustment for age and sex, inverse associations remained for systolic blood pressure, HbA1c, glucose level, diabetes mellitus, smoking, and obesity. This pattern was preserved after additional adjustment for systolic blood pressure.

**Table 6:** Quantitative impact of CV risk factors on global work efficiency (GWE), measured in %.

	Univariable model		Interaction with sex	Adjusted for age and sex		Adjusted for age, sex, systolic blood pressure	
	Δ (95% CI)	*P* value	*P* value	Δ (95% CI)	*P* value	Δ (95% CI)	*P* value
Body mass index [kg/m^2^]	−0.02 (−0.04 to −0.002)	0.029	0.117	+0.003 (−0.02 to +0.02)	0.784	–	–
Systolic blood pressure [mmHg]	−0.02 (−0.03 to −0.02)	<0.001	0.125	−0.01 (−0.02 to −0.005)	<0.001	–	–
Total cholesterol [mg/dl]	−0.003 (−0.005 to 0)	0.021	0.869	0 (−0.003 to +0.002)	0.856	–	–
LDL cholesterol [mg/dl]	−0.002 (−0.004 to + 0.001)	0.080	0.769	0 (−0.002 to +0.003)	0.936	–	–
HbA1c [%]	−0.5 (−0.7 to −0.4)	<0.001	0.915	−0.2 (−0.4 to −0.04)	0.015	−0.2 (−0.4 to −0.02)	0.028
Glucose level [mmol/L]	−0.3 (−0.3 to −0.2)	<0.001	0.381	−0.1 (−0.2 to −0.03)	0.008	−0.1 (−0.2 to −0.02)	0.019
Glucose level after 2 h [mmol/L]	−0.04 (−0.1 to +0.02)	0.194	0.509	+0.02 (−0.04 to +0.08)	0.523	–	–
Diabetes	−0.8 (−1.1 to −0.5)	<0.001	0.742	−0.4 (−0.7 to −0.04)	0.028	–	–
Hypertension	−0.9 (−1.0 to −0.7)	<0.001	0.883	−0.5 (−0.6 to −0.3)	<0.001	–	–
Smoking	−0.2 (−0.5 to +0.001)	0.051	0.072	−0.4 (−0.6 to −0.1)	0.001	−0.4 (−0.6 to −0.2)	<0.001
Obesity	−0.4 (−0.7 to −0.2)	<0.001	0.944	−0.3 (−0.5 to −0.04)	0.020	−0.3 (−0.5 to −0.02)	0.038
Dyslipidaemia	−0.6 (−0.8 to −0.3)	<0.001	0.072	−0.08 (−0.3 to +0.2)	0.561	–	–

For interpretation of estimates and abbreviations please refer to legend of Table 3.

## Discussion

The current study investigated a well-characterized representative sample of the general population, aged 30–79 years, balanced for age and sex. We focused on the multifaceted associations of MyW, a novel echocardiographic approach quantifying active myocardial performance, with major CV risk factors and report three major findings. First, individuals exhibiting at least one risk factor had higher levels of all MyW domains except for work efficiency. This effect, compatible with a higher energy consumption during both the active systolic and early diastolic phase, was carried by an imbalanced increase in GWW resulting in lower work efficiency. Whereas GCW was higher in women compared to men, no such difference was found for GWW. Second, alterations in myocardial work were mainly induced by higher systolic blood pressure and pre-existing hypertension. In particular, hypertension showed the strongest association with all MyW domains and was the only risk factor with a pronounced and independent impact on GWW. Third, diabetes mellitus, obesity, dyslipidaemia, and smoking showed a pattern of isolated reduction in GCW and GWI, independent from systolic blood pressure, and did not affect GWW.

### Hypertension

Systemic hypertension is known to induce LV hypertrophy, and ultimately heart failure. Experimental models of human hypertensive hypertrophy in rats showed that myocardial efficiency decreases over time, and the disease aggravates from compensated hypertrophy to heart failure [21]. In a large cohort of hypertensive participants, LV hypertrophy was associated with depressed myocardial mechano-energetic efficiency which, in turn, predicted adverse outcome [22]. Previous analyses of our cohort [10] suggested a sex-specific sensitivity of the myocardium to individual CV risk factors, such as hypertension and dyslipidaemia. The present analysis showed an adverse effect of hypertension on the GWI in either sex, which consistently was stronger in women when compared to men. Further, previous studies in smaller groups of patients with hypertension reported an increase in GCW and GWW but found no changes in GWE [23, 24]. Along these lines, the NORRE consortium [25] also found GWI and GCW to be associated with systolic blood pressure. Our results confirm that hypertension was associated with higher constructive and wasted myocardial work, but revealed an adverse impact on work efficiency. A lower myocardial work efficiency, in line with lower myocardial mechano-energetic efficiency as described previously [22], might be the underlying pathomechanism driving symptomatic heart failure in hypertensive heart disease.

Systolic blood pressure and hypertension were the only risk factor studied enhancing wasted work. GWW by itself is very intriguing comprising the myocardium’s loss of energy during a heart cycle. This loss, according to Boe et al. [5], is an additional mechanical burden to the myocardium and may thus induce the accelerated development of heart failure. However, the exact mechanisms are not well understood. Loading conditions seem to interfere on wall tension by increasing wall stress and stiffness [26] and subsequently by increasing wasted work. This translates into increased myocardial oxygen demand and successively cardiac work augmentation. Lam et al. [27] found that in the early stages of hypertension, the antihypertensive therapy reduced arterial and LV systolic stiffness and lowered the ventricular-arterial coupling ratio. Thus, although total cardiac work was reduced, efficiency improved. We consistently found hypertension associated with higher total myocardial work but lower efficiency, which was explained by a disproportional increase of wasted work. Consequently, we would expect antihypertensive therapy to lower total myocardial work and optimize GWE by reducing GWW. Detailed assessment of hypertensive patients using echocardiography derived MyW indices might further enlighten the pathophysiology of hypertensive heart disease and identify potential treatment targets.

### Diabetes mellitus

Diabetes mellitus portends an increased risk for subsequent development of heart failure, and death [28]. Besides the development of CVD [28], diabetes mellitus seems also to directly affect the myocardium. Impaired GLS was found an early sign of diabetic heart disease [29], even in asymptomatic normotensive diabetes patients [30, 31], and was associated with adverse clinical outcomes [32]. In nondiabetic individuals, increased insulin resistance as a measure of glycaemic status was associated with impaired mechano-energetic efficiency [33]. In our study, diabetes mellitus and HbA1c were negatively associated with GCW and GWI. This association became even stronger after adjusting for systolic blood pressure, suggesting that diabetes and HbA1c affect systolic dysfunction independent of systolic blood pressure. After adjusting for blood pressure, we found an association of MyW efficiency with HbA1c but not with diabetes mellitus. MyW efficiency seems to be more affected by the level of chronic hyperglycemia as evidenced by HbA1c compared to the sole presence of diabetes mellitus. These findings emphasize the importance of enforcing blood sugar optimization to prevent cardiac damage in patients with diabetes mellitus.

### Obesity, dyslipidaemia, and smoking

Obesity, dyslipidaemia, and smoking are established risk factors for CV disease and heart failure [34–36]. Obesity, like diabetes mellitus, is associated with subtle systolic dysfunction [37]. Further, both entities are associated with poor glycaemic control and hyperinsulinemia, which may lead to changes in myocardial metabolism and fibrosis [38, 39]. Smoking is interrelated with inflammation, lipid abnormalities, and arterial stiffness which are prone to contribute to alterations in LV structure and function [40]. In our analysis, they revealed similar patterns of myocardial alteration by lowering GCW and GWI but without significant affection of GWW, independent from systolic blood pressure. Further, previous analyses of our cohort [10] found obesity adversely associated with myocardial deformation in either sex. In contrast, after adjustment for systolic blood pressure, myocardial work indices appeared more severely affected by obesity in women when compared to men. Further, these results strengthen the concept of sex-specific sensitivity of the myocardium to respective risk factors and should trigger further research in this field. The presence of CV risk factors in a sample of the general population free from heart failure seems to impact LV performance as assessed by MyW and reduce work efficiency through different pathways. MyW may potentially give new insights into the pathophysiology of different cardiac diseases, help to identify early abnormalities in LV function, and establish a more sensitive index for early stage dysfunction that opens the way to early preventive interventions.

### Strengths and limitations

To the best of our knowledge, this study is the first to provide a comprehensive assessment of the association of CV risk factors with echocardiography-based MyW indices, derived from a large, well-characterized representative sample of the general population. Within the STAAB cohort study, blood pressure was measured in a sitting position after 5 min of rest during the same 3-h study visit, in agreement with international recommendations [17]. Ideally, for this analysis, blood pressure should have been measured during the echocardiographic examination. Hypertension as a major risk factor, had a key impact in this analysis, given its high prevalence and the haemodynamic impact on LV function, performance, and contractility. MyW method relies on the indirect measurement of brachial cuff pressure as a surrogate of invasively measured pressure, which might affect the accuracy of the estimation of the various work domains. However, the approach used in the present study was reported to yield good agreement with invasive measurements [1].

## Conclusion

Individual CV risk factors selectively impact on constructive and wasted active myocardial function as measured by MyW domains. In particular, the heart in hypertension appears to operate at higher energy levels as indicated by both increased GCW and GWW, which results in lower work efficiency. This pathomechanism may drive the development of symptomatic heart failure in hypertensive heart disease. The other CV risk factors also adversely impact on GWE, predominantly by reducing GCW independent of systolic blood pressure. Quantifying active systolic and diastolic compromise by derivation of MyW holds promise to improve our understanding of pathophysiological processes in cardiac disease. Further studies are needed to evaluate the value of selected MyW parameters or a respective pattern of MyW indices to assess the current health status of an individual patient.

### Summary

#### What is known about the topic

- Left ventricular ejection fraction and longitudinal strain are load-dependent and might thus overestimate left ventricular dysfunction. The reference standard to quantify ventricular dysfunction requires invasive haemodynamic assessment.
- “Myocardial work” represents a novel validated approach, which allows quantifying active myocardial performance by means of non-invasively derived echocardiographic pressure–strain loops.
- The components and indices of myocardial work are viewed as reliable surrogates of appropriate or disproportionate myocardial energy consumption.

#### What this study adds

- Cardiovascular risk factors adversely affect myocardial work, independent from systolic blood pressure, individually, cumulatively, and in a sex-specific manner.
- Hypertension profoundly compromises myocardial work, in particular by increasing global wasted work. The heart in hypertension appears to operate at higher energy levels, yet lower efficiency.
- Quantifying active systolic and diastolic domains by means of myocardial work holds promise to improve our understanding of pathophysiological processes in cardiac disease.

## Supplementary information

Supplemental Data
