# Supplementary material for: Impact of cardiovascular risk factors on myocardial work—insights from the STAAB cohort study
Source: J Hum Hypertens. 2021 Mar 2;36(3):235–45. doi: 10.1038/s41371-021-00509-4 (PMC8930764; doi:10.1038/s41371-021-00509-4)
Supplement: Supplementary file 1 — Supplemental Data [file 41371_2021_509_MOESM1_ESM.docx]

**Supplemental Data**

**Impact of cardiovascular risk factors on myocardial work –**

**Insights from the STAAB cohort study**

Floran Sahiti MD^1,2^, Caroline Morbach MD^1,2^, Vladimir Cejka^1^, Theresa Tiffe MSc PhD^1,3^, Martin Wagner MD PhD^3^, Felizitas A Eichner MSc^1,3^, Götz Gelbrich PhD PhD^1,3,4^, Peter U Heuschmann MD MPH^1,3,4*^, Stefan Störk MD PhD^1,2*^

*both authors contributed equally

1 Comprehensive Heart Failure Center, University Hospital and University of Würzburg, Würzburg, Germany

2 Department of Medicine I, University Hospital of Würzburg, Würzburg, Germany

3 Institute of Clinical Epidemiology and Biometry, University of Würzburg, Würzburg, Germany

4 Clinical Trial Center, University Hospital of Würzburg, Würzburg, Germany

We thank the STAAB Consortium which consists of: S. Frantz (Dept. of Medicine I, Div. of Cardiology, University Hospital Würzburg); C. Maack (Comprehensive Heart Failure Center, University Hospital and University of Würzburg); G. Ertl (University Hospital Würzburg); M. Fassnacht (Dept. of Medicine I, Div. of Endocrinology, University Hospital Würzburg); C. Wanner (Dept. of Medicine I, Div. of Nephrology, University Hospital Würzburg); R. Leyh (Dept. of Cardiac & Thoracic Surgery, University Hospital Würzburg); J. Volkmann (Dept. of Neurology, University Hospital Würzburg); J. Deckert (Dept. of Psychiatry, Psychosomatics and Psychotherapy, Center of Mental Health, University Hospital Würzburg); H. Faller (Dept. of Medical Psychology, University of Würzburg); R. Jahns (Interdisciplinary Bank of Biomaterials and Data Würzburg, University Hospital Würzburg).

**Supplemental Data**

|  |  | **Intra-observer variability** | |  | **Inter-observer variability** | |
| --- | --- | --- | --- | --- | --- | --- |
|  | **Mean  (SD)** | **Mean  difference (SD)** | **95% CI of differences** |  | **Mean  difference (SD)** | **95% CI of differences** |
| **GCW** [mmHg%] | 2532 (472) | 26.4 (47.4) | 4.2; 48.5 |  | 231 (200) | 138; 325 |
| **GWW** [mmHg%] | 87.0 (44.2) | 7.7 (18.5) | –1.0; 16.3 |  | –9.9 (49.4) | –33.0; 13.2 |
| **GWI** [mmHg%] | 2269 (413) | 20.2 (52.1) | –4.2; 44.6 |  | 186 (152) | 115; 257 |
| **GWE** [%] | 95.5 (1.6) | –0.15 (0.49) | –0.38; –0.01 |  | 0.6 (1.86) | –0.2; 1.4 |

**Supplemental table S1**: Observer variability for parameters describing myocardial work.

To assess intra-observer variability, 20 random scans were read by one person twice (FS), more than 2 weeks apart.
To assess inter-observer variability, the same scans were read by a second person (CM) blinded to the previous results. GCW = global constructive work, GWW = global wasted work, GWE = global work efficiency, GWI= global work index, SD = standard deviation, CI = confidence interval

**Supplemental table S2.** Sensitivity analysis comparing subjects with feasible vs non-feasible myocardial work (MyW) derivation

|  | Total  sample  (n=2473) | MyW analysis  possible  (n=1929) | MyW not  possible  (n=544) | P-value |
| --- | --- | --- | --- | --- |
| Female sex | 1269 (51.3) | 951 (49.3) | 318 (58.4) | **<0.001** |
| Age, years | 54 (12) | 54 (12) | 56 (12) | **<0.001** |
| LV ejection fraction, % | 60 (5) | 61 (5) | 58 (5) | **<0.001** |
| e/e’ mean | 8 (3) | 8 (3) | 8 (3) | **<0.001** |
| LDL cholesterol, mg/dl | 123 (35) | 122 (34) | 124 (36) | 0.411 |
| HbA1c, % | 5.5 (0.6) | 5.6 (0.6) | 5.7 (0.7) | **<0.001** |
| eGFR, ml/min | 86 (15) | 87 (15) | 84 (16) | **0.002** |
| Heart rate, beats/min | 68 (10) | 67 (10) | 70 (12) | **<0.001** |
| Body mass index, kg/m^2^ | 27 (5) | 26 (4) | 28 (7) | **<0.001** |
| Diabetes mellitus | 238 (10) | 157 (8) | 81 (15) | **<0.001** |
| Hypertension | 1124 (45) | 849 (44) | 275 (51) | **0.007** |
| Smoking | 463 (18.7) | 376 (19.4) | 87 (15.9) | 0.069 |
| Obesity | 485 (19.6) | 302 (15.6) | 183 (33.6) | **<0.001** |
| Dyslipidaemia | 347 (14.0) | 255 (13.2) | 92 (16.9) | **0.005** |

Data are n (%) or mean (SD).

LV, left ventricular; LDL, low-density lipoprotein; eGFR, estimated glomerular filtration rate; HbA1c, glycosylated haemoglobin
